# Genetically modified crops are superior in their nitrogen use efficiency-A meta-analysis of three major cereals

**DOI:** 10.1038/s41598-020-65684-9

**Published:** 2020-05-22

**Authors:** Mengjiao Li, Jili Xu, Zhiyuan Gao, Hui Tian, Yajun Gao, Khalil Kariman

**Affiliations:** 1 Key Laboratory of Plant Nutrition and Agri-environment in Northwest China, Ministry of Agriculture, College of Natural Resources and Environment, Northwest A&F University, Yangling, Shaanxi China; 2 School of Agriculture and Environment, The University of Western Australia, Crawley, WA 6009 Australia

**Keywords:** Agricultural genetics, Molecular engineering in plants

## Abstract

It is currently uncertain to what extent genetic transformations of strategic crops (targeting diverse traits) have improved their N use efficiency (NUE), and what the key factors affecting their NUE are. Based on data collected from 130 publications, the effect sizes of genetic transformations and the key factors influencing NUE for three major cereal crops (rice, maize, and wheat), were investigated using a meta-analysis approach. Genetic transformations increased yield, shoot biomass, N uptake efficiency (NUpE), and partial factor productivity of N (PFPN) in the crops, but decreased shoot NUE (SNUE) and grain NUE (GNUE). Transporter genes improved yield and NUE parameters more efficiently, than did the other gene types. The effect sizes for some NUE parameters varied according to crop species and experimental conditions but did not differ between the overexpression and ectopic expression methods. Most effect sizes did not correlate with gene overexpression levels. These results indicate a promising potential of genetic transformations approaches for improving certain NUE parameters.

## Introduction

Nitrogen (N) is one of the most important nutrients for plant growth and is the second most important factor controlling plant productivity, after water deficiency^1^. In modern agriculture, high crop yields are generally associated with the application of large amounts of N fertilizers. More than 100 Tg N yr^−1^ of reactive N are industrially produced worldwide, and approximately 50% of these are applied in the cultivation of three major cereals, namely rice (*Oryza sativa*), maize (*Zea mays*), and wheat (*Triticum aestivum*)^2^, which collectively provide two-thirds of the world’s food energy intake^3^. However, in intensive farming systems, more N fertilizers than necessary are often applied. For instance, less than half of the N fertilizer used in China is taken up by crops^4^. The excess N is largely lost to the environment through a combination of leaching, surface run-off, denitrification, volatilization, and microbial consumption^5^; it subsequently causes various environmental issues such as air pollution^6^, soil acidification and degradation^7^, and water eutrophication^8^. To reduce the environmental burden of N fertilizers, it is required that N agricultural inputs are urgently reduced through diverse actions such as improving the N use efficiency (NUE) of crops. Moreover, it is estimated that a 1% increase in the NUE of crops could save approximately $1.1 billion annually^9^, which could be a considerable economic benefit.

NUE can be defined and quantified in a variety of manners^10^; however, N uptake efficiency (NUpE) (the capacity of plant roots to acquire N from the soil) and N utilization efficiency (NUtE) (the biomass or grain yield per unit of N uptake by the crop) are the main NUE parameters^11^. It is controversial which NUE factor (NUpE or NUtE) carries more weight when evaluating NUE^12^. Improved NUpE can lead to less excess N in agricultural soils and reduced environmental risks, while improved NUtE contributes to higher crop yields or biomass with less N input. Thus, both the NUpE and NUtE factors should be considered in NUE studies. Improving agricultural practices such as reducing N inputs based on the soil fertility status, practicing precision fertilization, and applying N fertilizers based on the growth stage of the crop are some useful approaches to improve NUE^13^. In China, the yields of rice, wheat, and maize were improved by 18.1%, 23.6%, and 35.2%, respectively, without any increase in N fertilizer application, by utilizing integrated soil-crop system management practices^14^.

Alternatively, the development of new crop varieties with high NUEs in different soil fertilization conditions, would be another way to improve NUE in agriculture. However, crop varieties from traditional breeding methods usually achieve high yields regardless of fertilizer input, and the selected varieties are not necessarily have high NUE^15^. Genetic transformation allows for the insertion of exogenous genes into the plant genome, thus generating novel varieties with specifically incorporated traits that cannot be achieved through conventional breeding strategies. Genes involved in biotic (such as insect, weed, and pathogen) and abiotic (mainly drought and salinity) stress resistance have been used to produce genetically modified (GM) crops; most of the existing GM crops deal with herbicide tolerance and insect resistance^16^. Although herbicide tolerant and insect resistant GM crops may play important roles in reducing the application of agrochemicals in agro-ecosystems; it is controversial or unclear whether these GM traits can improve yield or NUE of the respective crops^17^.

Over the past decades, several studies have focused on genes with possible important roles in improving the NUE of crops. N use in plants starts with the trans-membrane transport of N from the soil to the roots, which is mainly mediated by nitrate transporters (NRT) and ammonium transporters (AMT)^18,19^. Overexpression of NRTs has been found to increase shoot biomass, yield, or NUpE in rice^20,21^. Overexpression of a putative high-affinity NRT gene (*NpNRT2.1*) in *Nicotiana plumbaginifolia* significantly increased the NO_3_^−^ influx in transgenic plants^22^. The overexpression of AMT genes in rice increased ammonium uptake but impaired plant growth and development, probably because of the toxicity caused by the high ammonium concentrations in the transgenic plants^23,24^. Within plant cells, nitrate transported by NRTs is reduced first to nitrite by nitrate reductase and then to ammonium by nitrite reductase. Ammonium is then incorporated into organic molecules by the glutamine synthetase and glutamate synthase pathways^10^. N assimilatory enzymes have long been suggested to play important roles in governing the NUtE of crops. Overexpression of glutamine synthetase genes was shown to increase the NUtE of maize^25^, rice^26^, and wheat^27^. Overexpression of NADH-GOGAT genes improved the NUE of rice^28^ and tobacco^29^. However, overexpression of some other key enzymes such as nitrate reductase and nitrite reductase did not improve the NUE of crops^30,31^, suggesting that genes involved in N assimilation pathways might play different roles in modifying the NUE of plants. By contrast, some genes that are not directly involved in N assimilation can also influence plant NUE, significantly. For instance, alanine aminotransferase (AlaAT) is involved in the synthesis and degradation of alanine; however, overexpression of *AlaAT* genes were shown to significantly increase NUtE of some crops such as canola and rice^32,33^. The NUtE of plants may also be improved by the overexpression of other genes such as transcription factors (*Dof1* and *NAC*), pyruvate orthophosphate dikinase, and early nodulin genes^11^. The effects of genetic transformations on the NUE of plants may vary according to plant species, soil fertilization status, and target gene types. Null and even negative influences of genetic transformations on plant NUE have previously been documented^34,35^. Considering these previous controversial conclusions, a global evaluation, via meta-analysis, on the influences of genetic transformations on crop plant NUE is necessary. We have thoroughly examined the existing literature dealing with the genetic transformations of the three major cereal crops, its effects on plant performance, and the experimental conditions involved. Based on 130 publications, the present study aimed to investigate: (1) whether the three major GM cereals have improved NUE; (2) which NUE parameters can be improved by genetic transformations; and (3) how target gene types, gene expression level, and environmental conditions influence the effects of genetic transformations on the NUE of a given crop.

## Methods and materials

### Data compilation

We collected data from journal articles in which transgenic techniques such as gene overexpression and ectopic expression were used to produce GM rice, maize, and wheat varieties. For the meta-analysis, we searched the literature by using the search terms *Bacillus thuringiensis*/cp4/epsps/bar/gox/pat/drought/salt/saline AND yield/nitrogen AND rice/wheat/maize/corn in Web of Science, Google Scholar, and Scopus. Only the articles that included yield, shoot biomass, or N concentration data were selected, which totaled 130 (Supplementary Tables 1–6; Supplementary file 1). Data from the original paper’s figures were extracted by Getdata Graph Digitizer (http://getdata-graph-digitizer.com). For the papers in which gene expression levels were determined by semi-quantitative methods such as northern blot, gene expression data were extracted by the ImageJ software (https://imagej.nih.gov/ij/index.html). We collected yield, shoot biomass, and N concentration/content data, and calculated (1) shoot N utilization efficiency (SNUE, total shoot biomass produced per unit of N in shoot); (2) grain N utilization efficiency (GNUE, total grain yield produced per unit of N in shoot); and (3) partial factor productivity of N (PFPN, grain yield per unit of N applied in soil), where it was possible. NUpE was represented by the total shoot N uptake data. Standard deviation (SD) was used as the measure of variability and was obtained or calculated from the published measure of variance in each study if necessary. In a few of the studies where there was a lack of both SD and standard error (SE) values, we imputed SD using the coefficient of variation from all complete cases^36^. For each observation, we monitored crop species, target gene types and expression levels, experimental conditions, and environmental stress. The genes collected were classified into five categories (Biotic stress related protein (BSRP), Enzyme, Transcription factor, Transporter and Other genes) based on their encoded protein types (Supplementary Table 7). Experimental types were categorized as either “Field” or “Pot” and the genetic transformations methods were categorized as either “Ectopic expression” or “Over-expression.” Abiotic stresses such as drought, salinity, and nutrient deficiency were considered as “stress” where the term “stress” was clearly mentioned and investigated in the publications, and abiotic stress status was considered as a “non-stress” if it was not mentioned and investigated in the publications. Publications reporting biotic stressors like insects, weeds, or pathogens were considered as “stressed” or “non-stressed” if agrochemicals were applied or not applied, respectively. The stress status was considered as “na” if neither the abiotic or biotic stresses could be assessed from the publication (Supplementary Tables 1–6).

### Statistical analyses

The natural log of the response ratio (RR) was used as a metric of the effect size in the meta-analysis, log_e_ *R* = log_e_ (X_GM_/X_WT_), where X_GM_ and X_WT_ are the mean values for the GM lines and wild-type controls, respectively. A value of log_e_ *R* = 0 indicates that genetic transformations had no effect. The variance of log_e_ *R* for each study was calculated using the inverse of the pooled variance^37^. We calculated the mean effect size and generated 95% confidence intervals (CIs) using the random-effects model in Metafor^38^. Compared with the fixed-effects model, the random-effects model accounts for differences across studies assuming they do not share a common mean effect but that there is random variation among studies^39^. In this model, the between-study variance (τ^2^) was estimated using a restricted maximum likelihood method^40^. For bootstrapping, we used 4999 iterations. Effect sizes were considered significant if the 95% CI did not overlap with 0. The use of more than one observation within a study may have overrepresented an effect from studies with many observations. To test whether this was the case, we randomly chose one observation from each study and conducted the same analysis for those selected observations only. A mean effect size was calculated for this selected dataset similar to the mean effect size of the whole dataset, which demonstrates that over-representation of the effects from particular studies did not occur^41^. The mean effect sizes of each gene category, crop types, and environmental conditions were also calculated using random-effects models as described above. The relationship between gene overexpression levels and the effect sizes of yield, shoot biomass, and NUE parameters were explained using a random-effects quadratic regression model. The dependent variable for the regression was RR, weighted by the inverse of the total variance (within-study plus τ^2^) for each observation. The relative gene overexpression level for each observation was then treated as a continuous, independent variable. Only the data with positive effect sizes were analyzed.

## Results

### Impact of genetic transformations on yield, shoot biomass, and NUE parameters

**Figure 1:**
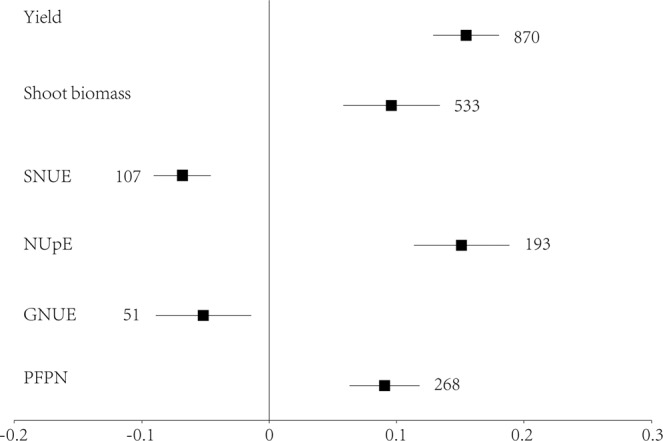
Effect sizes of yield, shoot biomass, shoot N utilization efficiency (SNUE), N uptake efficiency (NUpE), grain N utilization efficiency (GNUE) and partial factor productivity of N (PFPN). Error bars represent 95% bootstrapped confidence intervals (CIs). The effects of genetic transformations were considered significant if the 95% CI of the effect size did not overlap with zero. The number of observations for each category are shown next to the error bars.

Based on a total of 870 observations collated in our study, genetic transformations significantly increased the grain yield of crops (Fig. 1), and the effect size ranged from −2.11 to 2.75 (Supplementary Table 1). Among the total 870 observations, 648 showed positive effect sizes (Supplementary Table 1). The highest effect sizes of yield for rice, maize, and wheat occurred under stress conditions after ectopic expression of an ADP ribosylation factor 1 gene (*SaARF1*), a *Bacillus thuringiensis* (*Bt*) gene, and an *AlaAT* gene, respectively (Supplementary Table 1).

Genetic transformations increased shoot biomass of the crops based on a total of 533 observations (Fig. 1), and the effect sizes ranged from −2.87 to 1.94 (Supplementary Table 2). The largest effect sizes for rice, maize, and wheat were observed under stress conditions after ectopic expression of an *Aeluropus littoralis* stress-associated protein gene (*AISAP*), *Bt* gene, and a synthetic bacterial cold shock protein gene (*SeCspA*), respectively (Supplementary Table 2).

The NUpE of crops was also shown to be increased by genetic transformations (Fig. 1), and the effect sizes ranged from −1.00 to 1.30 (Supplementary Table 4). Overexpression of a glutamine synthetase gene in wheat and an NRT gene (*OsPTR6*) in rice, and ectopic expression of a *Bt* gene in maize were more effective at improving the NUpE of crops, when compared with the other genes (Supplementary Table 4). The PFPN was also significantly increased by genetic transformations (Fig. 1), and the effect sizes ranged from −1.27 to 0.78 (Supplementary Table 6). The greatest effect sizes for rice, maize, and wheat occurred following the overexpression of an NRT gene (*NRT1.1A*), and ectopic expression of a *Bt* gene and an *AlaAT* gene, respectively (Supplementary Table 6). However, genetic transformations significantly decreased the SNUE and GNUE parameters (Fig. 1).

Publication bias existed only for yield data (Egger’s test *p* < 0.001) (Supplementary Fig. 1). The heterogeneity for yield, shoot biomass, and NUE data was significant (*p* < 0.0001). Mean effect sizes were similar when we used one observation for each study (Supplementary Table 8), suggesting that the mean effect sizes were not overrepresented by studies with large numbers of observations.

### Influence of gene types and expression levels, and genetic transformation methods on effect sizes of yield, shoot biomass, and NUE parameters

**Figure 2:**
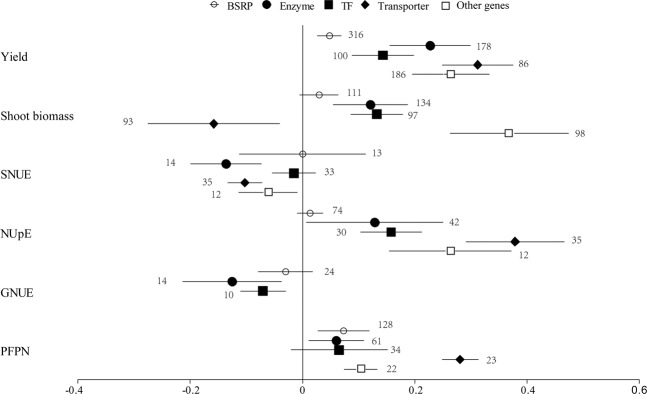
Effect sizes of yield, shoot biomass, shoot N utilization efficiency (SNUE), N uptake efficiency (NUpE), grain N utilization efficiency (GNUE) and partial factor productivity of N (PFPN) classified by gene categories. Only datasets with more than 10 observations were analyzed. BSRP: biotic stress related protein. TF: transcription factors. Error bars represent 95% bootstrapped confidence intervals (CIs). The effects of genetic transformation were considered significant if the 95% CIr of the effect size did not overlap with zero. The number of observations for each category are shown next to the error bars.

More than 80 genes were included in the collected data set (Supplementary Table 7). Only gene categories including more than 10 observations in yield, shoot biomass, and NUE parameters were selected for the meta-analysis dataset. Expression of genes encoding biotic stress-related protein (BSRP) significantly increased crop yield and PFPN, but did not influence shoot biomass, SNUE, NUpE, and GNUE (Fig. 2). Expression of genes encoding enzymes significantly improved yield, shoot biomass, NUpE, and PFPN, but decreased SNUE and GNUE. Expression of transcription factor genes also led to improved yield, shoot biomass, and NUpE, but did not influence SNUE and PFPN. Yield, NUpE, and PFPN parameters were improved upon expression of transporter genes, while shoot biomass and SNUE decreased. Expression of other genes such as late embryogenesis abundant protein and tiller inhibition gene also increased yield, shoot biomass, NUpE, and PFPN, but decreased SNUE. Taken together, transporter genes were the most effective ones for improving yield, NUpE, and PFPN compared to the other gene types, whereas the greatest effects on shoot biomass were found in crops with modified other genes (Fig. 2).

**Figure 3:**
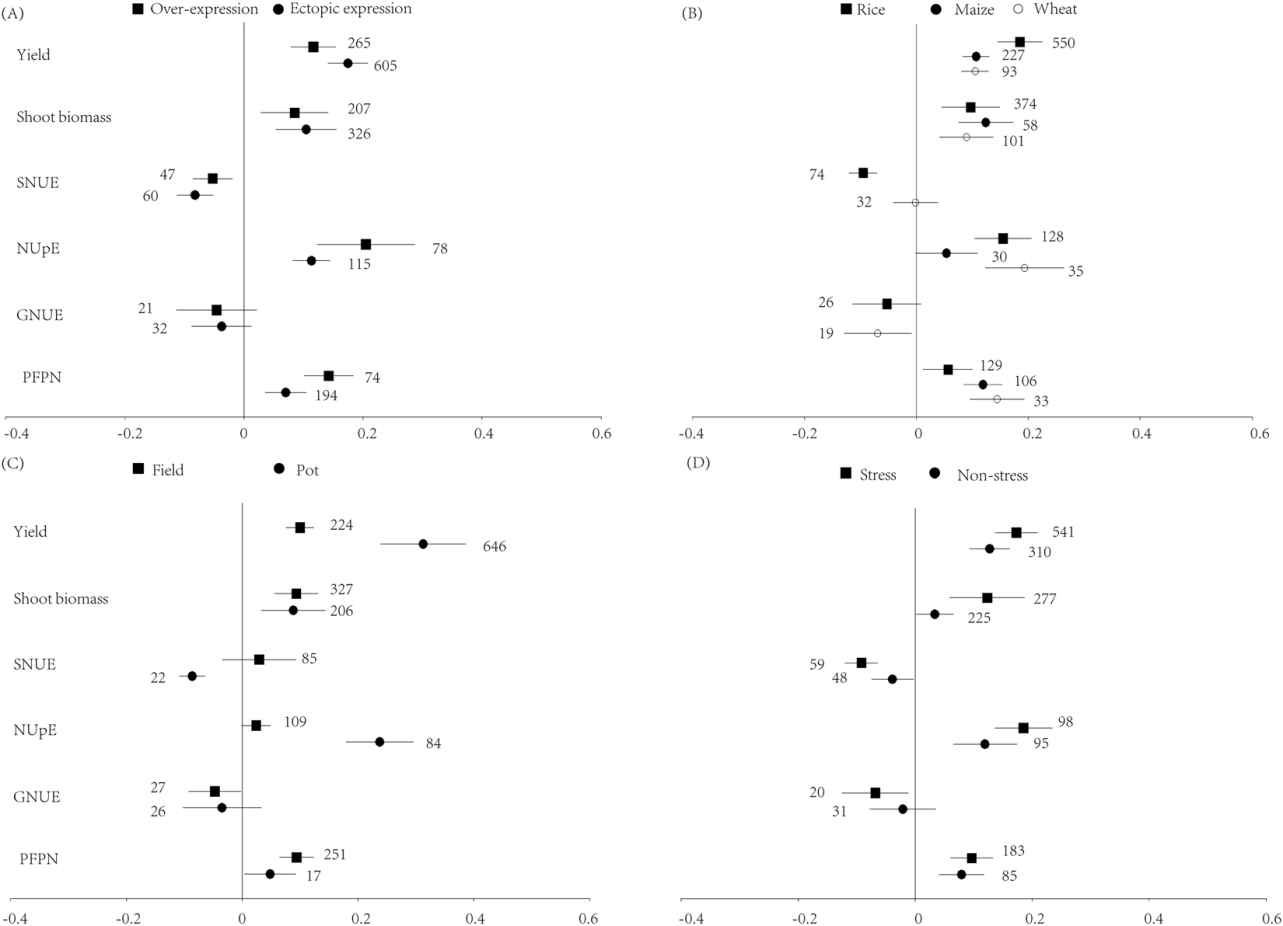
Effect sizes of yield, shoot biomass, shoot N utilization efficiency (SNUE), N uptake efficiency (NUpE), grain N utilization efficiency (GNUE) and partial factor productivity of N (PFPN) classified by transgenic types (**A**), crop species (**B**), experimental types (**C**) and environmental stress (**D**). The effects of genetic transformations were considered significant if the 95% CI of the effect size did not overlap with zero. The number of observations for each category are shown next to the error bars.

Both overexpression and ectopic expression increased yield, shoot biomass, NUpE, and PFPN, but did not influence GNUE and decreased SNUE. Overexpression and ectopic expression were not significantly different in influencing crop yield, shoot biomass and NUE indices (Fig. 3A).

**Figure 4:**
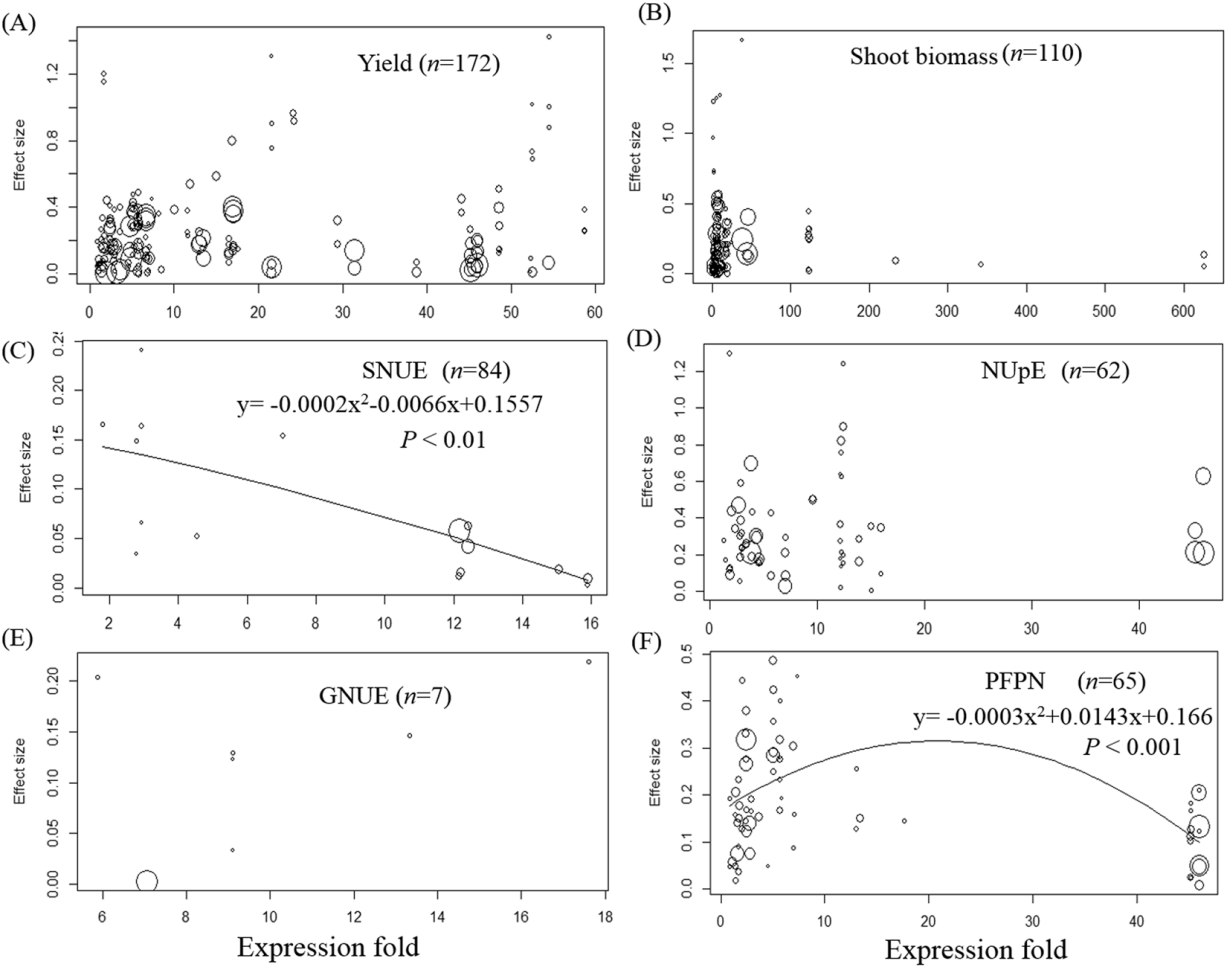
Relationship between effect sizes of yield, shoot biomass, shoot N utilization efficiency (SNUE), N uptake efficiency (NUpE), grain N utilization efficiency (GNUE), partial factor productivity of N (PFPN) and gene expression fold (gene expression level in transgenic plant/gene expression level in wild type plant). The size of each circle is proportional to that observation’s weight.

No correlation was observed between the magnitude of gene expression and the effect size of yields, shoot biomass, GNUE, and NUpE (Fig. 4A,B,D,E). However, the effect sizes of SNUE were negatively correlated with the magnitude of gene expression (Fig. 4C), and the relationship between the effect size of PFPN and the gene expression extent fitted a parabolic curve (Fig. 4F).

### Effect sizes of yield, shoot biomass, and NUE parameters as influenced by crop types and experimental conditions

Genetic transformations significantly improved yield, shoot biomass and PFPN for all the three crops, and the largest yield increase was in rice. SNUE of wheat was not influenced by genetic transformations, but it was decreased in rice. Genetic transformations increased NUpE of rice and wheat but did not influence the NUpE of maize. GNUE of rice was not influenced by genetic transformations, but in wheat it was decreased (Fig. 3B).

Genetic transformations increased yield, shoot biomass, and PFPN in both pot and field experiments, and the yield increase was greater in pot experiments than in field experiments. Genetic transformations did not influence SNUE in field experiments, but decreased SNUE in pot experiments. NUpE was increased by genetic transformations in pot experiments, but no impact was detected on NUpE in the field experiments. While genetic transformations did not influence GNUE in pot experiments, there was a negative effect on GNUE in field experiments (Fig. 3C).

Under both stressed and non-stressed conditions, genetic transformations increased yield, shoot biomass, NUpE, and PFPN, but decreased SNUE regardless of the environmental stress. Finally, GNUE of crops decreased with genetic transformations under stress conditions, whereas there was no significant impact on GNUE under non-stressed conditions (Fig. 3D).

## Discussion

Our meta-analysis indicated that genetic transformations significantly improved the yield of crops by 16.7% on average (Supplement Table 9). This means an additional 800 million people could be fed if all the planted rice, maize, and wheat in the world were GM plants, considering that these three crops provide two-thirds of the world’s food energy intake^3^. Publication bias existed for yield data because most observations (74.5%) indicated positive effect sizes. In other words, authors might not be motivated to publish their neutral or negative results from transgenic studies as positive data may be more easily accepted by journals. Genetic transformations increased crop yields may partly due to the higher shoot biomass and NUpE of GM crops. The increase of PFPN (9.47%) and NUpE (16.2%) (Supplement Table 9) suggests a higher crop yield could be obtained via genetic transformation approaches without enhancing N inputs, and consequently the environmental risks caused by N residues in soils may be reduced due to enhanced N uptake by GM crops. At least 4.7 Tg N could be saved each year if GM crops were planted as more than 50 Tg N are applied to the three major cereals each year globally^2^. Meanwhile, considerable amounts of fossil fuels could also be saved accordingly, as a result of the reduced demand for production and transport of N fertilizers. High NUE crop varieties may also be produced via conventional breeding approaches if controlled N fertilizer rates were applied; however, these approaches were mainly conducted in the presence of high fertilization inputs over the last 50 years^42^. Genetic transformations allow for the insertion of exogenous genes into the plant genome, which cannot be achieved through conventional breeding strategies and could have much more rapid and significant impacts on NUE. In addition, it is challenging to balance crop NUE and grain N content with conventional breeding methods because higher crop yields often lead to lower grain N content^43^. The negative relationship between yield and grain N content has been hypothesized due to competition between carbon and N for energy^44^ and/or, N dilution effects caused by carbon-based compounds^45^. N dilution effects may be alleviated by genetic transformations because GNUE was decreased by GM crops in our study, which means that genetic transformations could improve grain yields without grain N content loss. Although the safety of GM plants is still debated, for some developing countries with large populations and limited agricultural lands, the case for utilizing GM crops to help alleviate air pollution, water pollution, and soil acidification caused by the overuse of N fertilizers is becoming clearer^6,7^. Approximately 99% of the global GM crop acreage is related to insect- and herbicide-tolerance traits^17^ and these were traits targeted by most of the target genes analyzed in our study. Previous studies indicated that yield advantages of insect-resistant cotton in USA and China are less than 10% on average^46,47^, and yield effects are negligible for insect-resistant maize in the USA^48^. This is consistent with our findings, which indicated that the average yield increase for the three major GM cereals (with modified BSRP genes) are only 4.92% (Supplement Table 9). However, it is obvious that the yield effects of insect- or herbicide-tolerant GM crops largely depend on the intensity of the damage caused by the corresponding insect or weed. Transporter genes in the collected data mainly included NRT, sucrose transporter, potassium (K) transporter and Na^+^/H^+^ antiporter (NHX) genes, and the largest yield increase belonged to GM crops with modified NHX genes because these genes improved crop resistance to drought or salt stress^49^. Our study indicated that NRT genes had the largest impact of all transporter genes, and this may be because NRT genes mediate nitrate uptake by crop roots and are key drivers of NUE in crops^21,50,51^. Expression of transporter genes are not necessarily improve or even decreased shoot biomass. For example, expression of AMT genes led to an over-accumulation of ammonium, which may impair plant growth^23^. Expression of K transporters or NHX genes mainly improved shoot biomass under stress conditions^52,53^, and the neutral or negative effect sizes under normal conditions seem to reduce the overall effect sizes of these genes. Transcription factor genes are likely to ‘globally’ control the expression of a large numbers of genes that are involved in N metabolism, proteolytic enzymes, transporters and photosynthesis-related genes, and have significant potential as targets for NUE improvement^54^. However, among the five gene categories explored in our study, only transcription factor genes had no effect on PFPN, and the performance of transcription factor genes for improving other NUE parameters was not outstanding. This could be due to the fact that only a few transcription factors can significantly increase yield or NUE of crops, such as *ZmDOF1*, *AP37* and *OsNAC5*. Our study suggested that genes belonging to the category “other genes” should also be taken into consideration because some were efficient at improving yields or NUE parameters. For example, an ADP ribosylation factor 1 gene (*SaARF1*), which plays an important role in trafficking through the Golgi apparatus to the endoplasmic reticulum and from the trans-Golgi network to the endosome by interacting with COATOMER PROTEIN I (COPI) or COPII vesicle coat protein components^55^, was more efficient than other genes at improving crop yields under drought conditions.

Our study suggested that gene overexpression should be encouraged more than ectopic expression as this may reduce the potential ecological risks of GM plants^56^. In addition, the mere expression of the target genes can improve yield or NUE, regardless of the magnitude of gene expression. The widespread use of the 35S promoter of cauliflower mosaic virus (a strong constitutive promoter that can magnify gene expression to levels thousands of times higher than that in wild plants)^57^ in transgenic plants has been under fierce debate owing to its potential risks to the environment^58,59^. Considering the lack of correlation between the gene expression magnitude and NUE, we suggest that choosing the right target gene is more important than searching for highly efficient promoters, and the 35S promoter could be replaced by some weaker promoters to reduce the environmental risks of GM plants.

The effects of genetic transformation were different among the three crops for yield and some NUE parameters (Fig. 3B). Similar results have been also reported in previous studies. For example, overexpression of a glutamine synthetase gene in alfalfa did not influence NUE of the GM plant^60^, but overexpression of the same type gene in tobacco increased shoot biomass of the GM plant^61^. As a complex quantitative trait, NUE may be determined by multiple genes, and the contribution of the genes to NUE may vary among different crop species. This can be supported by the finding that genes included in the QTLs related to NUE were different among crop species^62,63^. Among the three crops, rice is the first whose genome sequencing were finished, and rice is easier to be transformed compared to maize and wheat^3^. Thus, more gene types have been introduced or overexpressed in rice, and this may explain the higher yield effect size of rice than maize and wheat in our study. In addition, gene segmental and tandem duplication events were more common in tetraploid or hexaploid plant species than in diploid plants^64^, which may decrease the effects of genetic transformations.

The effects of genetic transformations on crop yield, NUpE, and GNUE may be overestimated in pot experiments than in field experiments, and this is possibly because experimental conditions can be controlled more accurately in the former. The overestimation of NUpE may also contribute to the reduced SNUE observed in pot experiments. Positive effect sizes for yield, shoot biomass, and NUpE under non-stress conditions may be due to: (1) most of our data being obtained from under field conditions, and crops often suffer from environmental stresses more or less evenly under normal field conditions, and thus, GM crops may still benefit from the expression of the genes involved in the stress resistance under normal field conditions; and (2) expression of genes involved in biotic stress resistance could also improve plant growth under non-stress conditions^65^.

## Conclusions

We conclude that genetic transformations increased yield, shoot biomass, NUpE, and PFPN of crops, but decreased SNUE and GNUE. Transporter gene expression is more efficient at improving yields or NUE parameters of crops than are other type of genes. Genetic transformations improved the yields of rice more than that of maize and wheat. Yield and NUE parameters were enhanced more in pot experiments than in field experiments. To alleviate potential environmental risks of genetic transformations, gene overexpression using gentle promoters is recommended rather than ectopic expression or overexpression using high efficiency promoters, because the effect sizes of NUE parameters did not differ between the two genetic transformations methods and most effect sizes did not correlate with the extent of gene magnification. Genetic transformations improved yield, shoot biomass, and NUE parameters of crops under both normal and stress conditions.

## Supplementary information

Supplementary File 1.

Supplementary Table 1-6.

Supplementary Table 7.

Supplementary Table 8.

Supplementary Table 9.

Supplementary Fig. 1.
