# Supplementary material for: Genetically modified crops are superior in their nitrogen use efficiency-A meta-analysis of three major cereals
Source: Sci Rep. 2020 May 22;10:8568. doi: 10.1038/s41598-020-65684-9 (PMC7244766; doi:10.1038/s41598-020-65684-9)
Supplement: Supplementary file 1 [file 41598_2020_65684_MOESM1_ESM.pdf]

Genetically modified crops are superior in their nitrogen use efficiency-A meta-analysis of three major cereals

Mengjiao Li [1], Jili Xu [1], Zhiyuan Gao [1], Hui Tian [1,*], Yajun Gao [1,*] & Khalil Kariman[2]

[1] Key Laboratory of Plant Nutrition and Agri-environment in Northwest China, Ministry of Agriculture, College of Natural Resources and Environment, Northwest A&F University, Yangling, Shaanxi, China

[2] School of Agriculture and Environment, The University of Western Australia, Crawley, WA 6009, Australia

Corresponding authors:

1. Hui Tian, tianh@nwsuaf.edu.cn
2. Yajun Gao, yajungao@nwafu.edu.cn

References during meta-analysis

Amin M, Elias SM, Hossain A, Ferdousi A, Rahman MS, Tuteja N, Seraj ZI (2012) Over-expression of a DEAD-box helicase, PDH45, confers both seedling and reproductive stage salinity tolerance to rice (Oryza sativa L.). Molecular Breeding 30: 345-354. doi: 10.1007/s11032-011-9625-3.

Andersen MN, Sausse C, Lacroix B, Caul S, Messean A (2007) Agricultural studies of GM maize and the field experimental infrastructure of ECOGEN. Pedobiologia 51: 175-184. doi: 10.1016/j.pedobi.2007.03.005.

Bae MJ, Kim YS, Kim IS, Choe YH, Lee EJ, Kim YH, Park HM, Yoon HS (2013) Transgenic rice overexpressing the Brassica juncea gamma-glutamylcysteine synthetase gene enhances tolerance to abiotic stress and improves grain yield under paddy field conditions. Molecular Breeding 31: 931-945. doi: 10.1007/s11032-013-9846-8.

Bahieldin A, Mahfouz HT, Eissa HF, Saleh OM, Ramadan AM, Ahmed IA, Dyer WE, El-Itriby HA, Madkour MA (2005) Field evaluation of transgenic wheat plants stably expressing the HVA1 gene for drought tolerance. Physiologia Plantarum 123: 421-427. doi: 10.1111/j.1399-3054.2005.00470.x.

Baisakh N, RamanaRao MV, Rajasekaran K, Subudhi P, Janda J, Galbraith D, Vanier C, Pereira A (2012) Enhanced salt stress tolerance of rice plants expressing a vacuolar H plus -ATPase subunit c1 (SaVHAc1) gene from the halophyte grass Spartina alterniflora Loisel. Plant Biotechnology Journal 10: 453-464. doi: 10.1111/j.1467-7652.2012.00678.x.

Barro F, Barcelo P, Lazzeri PA, Shewry PR, Martin A, Ballesteros J (2002) Field evaluation and agronomic performance of transgenic wheat. Theoretical and Applied Genetics 105: 980-984. doi: 10.1007/s00122-002-0996-z.

Ben Saad R, Fabre D, Mieulet D, Meynard D, Dingkuhn M, Al-Doss A, Guiderdoni E, Hassairi A (2012) Expression of the Aeluropus littoralis AlSAP gene in rice confers broad tolerance to abiotic stresses through maintenance of photosynthesis. Plant Cell and Environment 35: 626-643. doi: 10.1111/j.1365-3040.2011.02441.x.

Bi YM, Kant S, Clark J, Gidda S, Ming F, Xu JY, Rochon A, Shelp BJ, Hao LX, Zhao R, Mullen RT, Zhu T, Rothstein SJ (2009) Increased nitrogen-use efficiency in transgenic rice plants over-expressing a nitrogen-responsive early nodulin gene identified from rice expression profiling. Plant Cell and Environment 32: 1749-1760. doi: 10.1111/j.1365-3040.2009.02032.x.

Biswas S, Amin USM, Sarker S, Rahman MS, Amin R, Karim R, Tuteja N, Seraj ZI (2018) Introgression, generational expression and salinity tolerance conferred by the Pea DNA helicase 45 transgene into two commercial rice genotypes, BR28 and BR47. Molecular Biotechnology 60: 111-123. doi: 10.1007/s12033-017-0055-2.

Brauer EK, Rochon A, Bi Y-M, Bozzo GG, Rothstein SJ, Shelp BJ (2011) Reappraisal of nitrogen use efficiency in rice overexpressing glutamine synthetase1. Physiologia Plantarum 141: 361-372. doi: 10.1111/j.1399-3054.2011.01443.x.

Bruns HA, Abbas HK (2006) Planting date effects on Bt and non-Bt corn in the Mid-South USA. Agronomy Journal 98: 100-106. doi: 10.2134/agronj2005.0143.

Bundo M, Coca M (2016) Enhancing blast disease resistance by overexpression of the calcium-dependent protein kinase OsCPK4 in rice. Plant Biotechnology Journal 14: 1357-1367. doi: 10.1111/pbi.12500.

Cai HM, Zhou Y, Xiao JH, Li XH, Zhang QF, Lian XM (2009) Overexpressed glutamine synthetase gene modifies nitrogen metabolism and abiotic stress responses in rice. Plant Cell Reports 28: 527-537. doi: 10.1007/s00299-008-0665-z.

Casaretto JA, El-kereamy A, Zeng B, Stiegelmeyer SM, Chen X, Bi YM, Rothstein SJ (2016) Expression of OsMYB55 in maize activates stress-responsive genes and enhances heat and drought tolerance. BMC Genomics 17. doi: 10.1186/s12864-016-2659-5.

Cha ZP, Wan BL, Du XS, Yin DS, Qi HX (2014) Analyzing of nitrogen use efficiency of NAT3 transgenci rice. Hubei Agricultural Science 53: 5653-5656.

Chen DD, Richardson T, Chai SC, McIntyre CL, Rae AL, Xue GP (2016a) Drought-Up-Regulated TaNAC69-1 is a transcriptional repressor of TaSHY2 and TaIAA7, and enhances root length and biomass in wheat. Plant and Cell Physiology 57: 2076-2090. doi: 10.1093/pcp/pcw126.

Chen G, Li CL, Gao ZY, Zhang Y, Zhu L, Hu J, Ren DY, Xu GH, Qian Q (2018a) Driving the expression of RAA1 with a drought-responsive promoter enhances root growth in rice, its accumulation of potassium and its tolerance to moisture stress. Environmental and Experimental Botany 147: 147-156. doi: 10.1016/j.envexpbot.2017.12.008.

Chen G, Liu CL, Gao ZY, Zhang Y, Jiang HZ, Zhu L, Ren DY, Yu L, Xu GH, Qian Q (2017) OsHAK1, a high-affinity potassium transporter, positively regulates responses to drought stress in rice. Frontiers in Plant Science 8. doi: ARTN 188510.3389/fpls.2017.01885.

Chen G, Liu CL, Gao ZY, Zhang Y, Zhang AP, Zhu L, Hu J, Ren DY, Yu L, Xu GH, Qian Q (2018b) Variation in the abundance of OsHAK1 transcript underlies the differential salinity tolerance of an indica and a japonica rice cultivar. Frontiers in Plant Science 8. doi: ARTN 221610.3389/fpls.2017.02216.

Chen GH, Hooks CRR, Patton TW, Kratochvil R, Dively G (2016b) Tolerance to stalk and ear-invading worms and yield performance of Bt and conventional corn hybrids. Agronomy Journal 108: 73-84. doi: 10.2134/agronj15.0139.

Chen XJ, Chen Y, Zhang LN, Xu B, Zhang JH, Chen ZX, Tong YH, Zuo SM, Xu JY (2016c) Overexpression of OsPGIP1 enhances rice resistance to sheath blight. Plant Disease 100: 388-395. doi: 10.1094/Pdis-03-15-0305-Re.

Chen YS, Lo SF, Sun PK, Lu CA, Ho THD, Yu SM (2015) A late embryogenesis abundant protein HVA1 regulated by an inducible promoter enhances root growth and abiotic stress tolerance in rice without yield penalty. Plant Biotechnology Journal 13: 105-116. doi: 10.1111/pbi.12241.

Cho JI, Lim HM, Siddiqui ZS, Park SH, Kim AR, Kwon TR, Lee SK, Park SC, Jeong MJ, Lee GS (2014) Over-expression of PsGPD, a mushroom glyceraldehyde-3-phosphate dehydrogenase gene, enhances salt tolerance in rice plants. Biotechnology Letters 36: 1641-1648. doi: 10.1007/s10529-014-1522-5.

Choe YH, Kim YS, Kim IS, Bae MJ, Lee EJ, Kim YH, Park HM, Yoon HS (2013) Homologous expression of gamma-glutamylcysteine synthetase increases grain yield and tolerance of transgenic rice plants to environmental stresses. Journal of Plant Physiology 170: 610-618. doi: 10.1016/j.jplph.2012.12.002.

Dey A, Samanta MK, Gayen S, Maiti MK (2016) The sucrose non-fermenting 1-related kinase 2 gene SAPK9 improves drought tolerance and grain yield in rice by modulating cellular osmotic potential, stomatal closure and stress-responsive gene expression. BMC Plant Biology 16. doi: ARTN 158 10.1186/s12870-016-0845-x.

Dillehay BL, Roth GW, Calvin DD, Kratochvil RJ, Kuldau GA, Hyde JA (2004) Performance of Bt

corn hybrids, their near isolines, and leading corn hybrids in Pennsylvania and Maryland. Agronomy Journal 96: 818-824. doi: 10.2134/agronj2004.0818.

Ding ZS, Huang SH, Zhou BY, Sun XF, Zhao M (2013) Over-expression of phosphoenolpyruvate carboxylase cDNA from C4 millet (Seteria italica) increase rice photosynthesis and yield under upland condition but not in wetland fields. Plant Biotechnology Reports 7: 155-163. doi: 10.1007/s11816-012-0244-1.

Estes RE, Tinsley NA, Gray ME (2016) Evaluation of soil-applied insecticides with Bt maize for managing corn rootworm larval injury. Journal of Applied Entomology 140: 19-27. doi: 10.1111/jen.12233.

Fan XR, Xie D, Chen JG, Lu HY, Xu YL, Ma C, Xu GH (2014) Over-expression of OsPTR6 in rice increased plant growth at different nitrogen supplies but decreased nitrogen use efficiency at high ammonium supply. Plant Science 227: 1-11. doi: 10.1016/j.plantsci.2014.05.013.

Fang ZM, Bai GX, Huang WT, Wang ZX, Wang XL, Zhang MY (2017) The rice peptide transporter OsNPF7.3 is induced by organic nitrogen, and contributes to nitrogen allocation and grain yield. Frontiers in Plant Science 8. doi: 10.3389/fpls.2017.01338.

Feng HM, Lu H, Wang HQ, Li XY (2017) Function analyses of rice nitrate transporter gene OsNPF7.9 in nitrogen accumulation and transport. Chinese Journal of Rice Science 31: 457-464.

Graeber JV, Nafziger ED, Mies DW (1999) Evaluation of transgenic, Bt-containing corn hybrids. Journal of Producttion Agriculture 12: 659-663. doi: 10.2134/jpa1999.0659.

Habash DZ, Massiah AJ, Rong HL, Wallsgrove RM, Leigh RA (2001) The role of cytosolic glutamine synthetase in wheat. Annals of Applied Biology 138: 83-89. doi: 10.1111/j.1744-7348.2001.tb00087.x.

Haegele JW, Below FE (2013) Transgenic corn rootworm protection increases grain yield and nitrogen use of maize. Crop Science 53: 585-594. doi: 10.2135/cropsci2012.06.0348.

He X, Qu B, Li W, Zhao X, Teng W, Ma W, Ren Y, Li B, Li Z, Tong Y (2015) The nitrate-inducible NAC transcription factor TaNAC2-5A controls nitrate response and increases wheat yield. Plant Physiology 169: 1991-2005. doi: 10.1104/pp.15.00568.

Helliwell EE, Wang Q, Yang YN (2013) Transgenic rice with inducible ethylene production exhibits broad-spectrum disease resistance to the fungal pathogens Magnaporthe oryzae and Rhizoctonia solani. Plant Biotechnology Journal 11: 33-42. doi: 10.1111/pbi.12004.

Hong YB, Yang YY, Zhang HJ, Huang L, Li DY, Song FM (2017) Overexpression of MoSM1, encoding for an immunity-inducing protein from Magnaporthe oryzae, in rice confers broad-spectrum resistance against fungal and bacterial diseases. Scientific Reports 7. doi: ARTN 4103710.1038/srep41037.

Hoque MS, Masle J, Udvardi MK, Ryan PR, Upadhyaya NM (2006) Over-expression of the rice OsAMT1-1 gene increases ammonium uptake and content, but impairs growth and development of plants under high ammonium nutrition. Functional Plant Biology 33: 153-163. doi: 10.1071/fp05165.

Hu HH, Dai MQ, Yao JL, Xiao BZ, Li XH, Zhang QF, Xiong LZ (2006) Overexpressing a NAM, ATAF, and CUC (NAC) transcription factor enhances drought resistance and salt tolerance in rice. Proceedings of the National Academy of Sciences of the United States of America 103: 12987-12992. doi: 10.1073/pnas.0604882103.

Huang Y, Li JK, Qiang S, Dai WM, Song XL (2016) Transgenic restorer rice line T1c-19 with stacked cry1C*/bar genes has low weediness potential without selection pressure. Journal of

Integrative Agriculture 15: 1046-1058. doi: 10.1016/S2095-3119(15)61219-9.

Huang YM, Xiao BZ, Xiong LZ (2007) Characterization of a stress responsive proteinase inhibitor gene with positive effect in improving drought resistance in rice. Planta 226: 73-85. doi: 10.1007/s00425-006-0469-8.

Hussain A, Shahzad A, Tabassum S, Hafeez H, Khattak JZK (2018) Salt stress tolerance of transgenic rice (Oryza sativa L.) expressing AtDREB1A gene under inducible or constitutive promoters. Biologia 73: 31-41. doi: 10.2478/s11756-018-0010-0.

Islam SMT, Tammi RS, Singla-Pareek SL, Seraj ZI (2010) Enhanced salinity tolerance and improved yield properties in Bangladeshi rice Binnatoa through Agrobacterium-mediated transformation of PgNHX1 from Pennisetum glaucum. Acta Physiologiae Plantarum 32: 657-663. doi: 10.1007/s11738-009-0443-8.

Jeong JS, Kim YS, Redillas M, Jang G, Jung H, Bang SW, Choi YD, Ha SH, Reuzeau C, Kim JK (2013) OsNAC5 overexpression enlarges root diameter in rice plants leading to enhanced drought tolerance and increased grain yield in the field. Plant Biotechnology Journal 11: 101-114. doi: 10.1111/pbi.12011.

Jiang Y, Huang SQ, Cai ML, Li CF, Kong X, Zhang F, Mohamed I, Cao CG (2013) Yield changes of Bt-MH63 with cry1C* or cry2A* genes compared with MH63 (Oryza sativa) under different nitrogen levels. Field Crops Research 151: 101-106. doi: 10.1016/j.fcr.2013.06.017.

Jiang Y, Ling L, Zhang LL, Domingo A, Cai ML, Li CF, Zhan M, Wang JP, Cao CG (2017) Different response of an elite Bt restorer line of hybrid rice (Oryza sativa L.) in adaptation to nitrogen deficiency. Acta Physiologiae Plantarum 39. doi: ARTN 8910.1007/s11738-017-2384-y.

Jiang Y, Ling L, Zhang LL, Wang KX, Cai ML, Zhan M, Li CF, Wang JP, Chen X, Lin YJ, Cao CG (2016) Transgenic Bt (Cry1Ab/Ac) rice lines with different genetic backgrounds exhibit superior field performance under pesticide-free environment. Field Crops Research 193: 117-122. doi: 10.1016/j.fcr.2016.03.014.

Jiang Y, Ling L, Zhang LL, Wang KX, Li XX, Cai ML, Zhan M, Li CF, Wang JP, Cao CG (2018) Comparison of transgenic Bt rice and their non-Bt counterpart in yield and physiological response to drought stress. Field Crops Research 217: 45-52. doi: 10.1016/j.fcr.2017.12.007.

Jiang Y, Meng JJ, Zhang LL, Cai ML, Li CF, Zhan M, Wang JP, Wang BF, Mohamed I, Cao CG (2014) Non-target effects of Bt transgenes on grain yield and related traits of an elite restorer rice line in response to nitrogen and potassium applications. Field Crops Research 169: 39-48. doi: 10.1016/j.fcr.2014.09.011.

Joshi R, Ramanarao MV, Lee S, Kato N, Baisakh N (2014) Ectopic expression of ADP ribosylation factor 1 (SaARF1) from smooth cordgrass (Spartina alterniflora Loisel) confers drought and salt tolerance in transgenic rice and Arabidopsis. Plant Cell Tissue and Organ Culture 117: 17-30. doi: 10.1007/s11240-013-0416-x.

Katayama H, Mori M, Kawamura Y, Tanaka T, Mori M, Hasegawa H (2009) Production and characterization of transgenic rice plants carrying a high-affinity nitrate transporter gene (OsNRT2.1). Breeding Science 59: 237-243. doi: DOI 10.1270/jsbbs.59.237.

Kim S, Kim C, Li W, Kim T, Li Y, Zaidi MA, Altosaar I (2008) Inheritance and field performance of transgenic Korean Bt rice lines resistant to rice yellow stem borer. Euphytica 164: 829-839. doi: 10.1007/s10681-008-9739-9.

Kim YS, Kim IS, Choe YH, Bae MJ, Shin SY, Park SK, Kang HG, Kim YH, Yoon HS (2014) Overexpression of the Arabidopsis vacuolar H+-pyrophosphatase AVP1 gene in rice plants

improves grain yield under paddy field conditions. The Journal of Agricultural Science 152: 941-953. doi: 10.1017/S0021859613000671.

Kocourek F, Stara J (2012) Efficacy of Bt Maize against European Corn Borer in Central Europe. Plant Protect Science 48: S25-S35.

Kumar A, Kaiser BN, Siddiqi MY, Glass ADM (2006) Functional characterisation of OsAMT1.1 overexpression lines of rice, Oryza sativa. Functional Plant Biology 33: 339-346. doi: 10.1071/Fp05268.

Kumar M, Choi J, An G, Kim SR (2017) Ectopic expression of OsSta2 enhances salt stress tolerance in rice. Frontiers in Plant Science 8. doi: ARTN 31610.3389/fpls.2017.00316.

Kumar M, Lee SC, Kim JY, Kim SJ, Aye SS, Kim SR (2014) Over-expression of dehydrin gene, OsDhn1, improves drought and salt stress tolerance through scavenging of reactive oxygen species in rice (Oryza sativa L.). Journal of Plant Biology 57: 383-393. doi: 10.1007/s12374-014-0487-1.

Kurai T, Wakayama M, Abiko T, Yanagisawa S, Aoki N, Ohsugi R (2011) Introduction of the ZmDof1 gene into rice enhances carbon and nitrogen assimilation under low-nitrogen conditions. Plant Biotechnology Journal 9: 826-837. doi: 10.1111/j.1467-7652.2011.00592.x.

Lauer J, Wedberg J (1999) Grain yield of initial Bt corn hybrid introductions to farmers in the northern corn belt. Journal of Production Agriculture 12: 373-376. doi: DOI 10.2134/jpa1999.0373.

Leaf T, Ostlie K, Kaiser D (2017) Transgenic corn response to nitrogen rates under corn rootworm pressure. Agronomy Journal 109: 1632-1641. doi: 10.2134/agronj2016.03.0154.

Lian L, Wang XW, Zhu YS, He W, Cai QH, Xie HA, Zhang MQ, Zhang JF (2014) Physiological and photosynthetic characteristics of indica Hang2 expressing the sugarcane PEPC gene. Molecular Biology Reports 41: 2189-2197. doi: 10.1007/s11033-014-3070-4.

Ling F, Zhou F, Chen H, Lin YJ (2016) Development of marker-free insect-resistant indica rice by agrobacterium tumefaciens-mediated co-transformation. Frontiers in Plant Science 7. doi: 10.3389/fpls.2016.01608.

Liu WQ, Meng QC, Weng LS, Peng J, Xiao YL, Yu JH, Yi ZL, Xiao GY (2016) A comparative study of two-line early season hybrid rice with lepidopteran resistance. Field Crops Research 187: 107-112. doi: 10.1016/j.fcr.2015.12.014.

Lu HYL, SY, Tang Z, Xu GH, Fan XR (2015) Over-expressing OsNRT2.3b in rice Wuyujing 7 can promote rice growth and enhance grain yield. Molecular Plant Breeding 13: 497-504.

Lu Y, Li YJ, Zhang JC, Xiao YT, Yue YS, Duan LS, Zhang MC, Li ZH (2013) Overexpression of arabidopsis molybdenum cofactor sulfurase gene confers drought tolerance in maize (Zea mays L.). Plos One 8. doi: ARTN e5212610.1371/journal.pone.0052126.

Ma BL, Meloche F, Wei L (2009) Agronomic assessment of Bt trait and seed or soil-applied insecticides on the control of corn rootworm and yield. Field Crops Research 111: 189-196. doi: 10.1016/fcr.2008.12.006.

Ma BL, Subedi KD (2005) Development, yield, grain moisture and nitrogen uptake of Bt corn hybrids and their conventional near-isolines. Field Crops Research 93: 199-211. doi: 10.1016/j.fcr.2004.09.021.

Ma C, Fan XR, Xu GH (2011) Responses of rice plants of Wuyunjing 7 to nitrate as affected by over-expression of OsNRT1.2. Chinese Journal of Rice Science 25: 349-356.

Magg T, Melchinger AE, Klein D, Bohn M (2001) Comparison of Bt maize hybrids with their non-transgenic counterparts and commercial varieties for resistance to European corn borer

and for agronomic traits. Plant Breeding 120: 397-403. doi: 10.1046/j.1439-0523.2001.00621.x.

Michelotto MD, Neto JC, Pirotta MZ, Duarte AP, de Feitas RS, Finoto EL (2017) Efficacy of transgenic mayze insecticide treatment to control fall armyworm in late-season maize in Sao Paulo state, Brazil. Ciencia E Agrotecnologia 41: 128-138. doi: 10.1590/1413-70542017412020816.

Moin M, Bakshi A, Madhav MS, Kirti PB (2017) Expression profiling of ribosomal protein gene family in dehydration stress responses and characterization of transgenic rice plants overexpressing RPL23A for water-use efficiency and tolerance to drought and salt stresses. Frontiers in Chemistry 5. doi: ARTN 9710.3389/fchem.2017.00097.

Mungai NW, Motavalli PP, Nelson KA, Kremer RJ (2005) Differences in yields, residue composition and N mineralization dynamics of Bt and non-Bt maize. Nutrient Cycling in Agroecosystems 73: 101-109. doi: 10.1007/s10705-005-8850-8.

Nakashima K, Tran LSP, Van Nguyen D, Fujita M, Maruyama K, Todaka D, Ito Y, Hayashi N, Shinozaki K, Yamaguchi-Shinozaki K (2007) Functional analysis of a NAC-type transcription factor OsNAC6 involved in abiotic and biotic stress-responsive gene expression in rice. Plant Journal 51: 617-630. doi: 10.1111/j.1365-313X.2007.03168.x.

Novacek MJ, Mason SC, Galusha TD, Yaseen M (2014) Bt transgenes minimally influence maize grain yield and lodging across plant populations. Maydica 59: 91-96.

Nuccio ML, Wu J, Mowers R, Zhou HP, Meghji M, Primavesi LF, Paul MJ, Chen X, Gao Y, Haque E, Basu SS, Lagrimini LM (2015) Expression of trehalose-6-phosphate phosphatase in maize ears improves yield in well-watered and drought conditions. Nature Biotechnology 33: 862-869. doi: 10.1038/nbt.3277.

Obopile M, Hammond RB, Thomison PR (2013) Interaction among planting dates, transgenic maize, seed treatment, corn rootworm damage and grain yield. Journal of Applied Entomology 137: 45-55. doi: 10.1111/j.1439-0418.2012.01716.x.

Oh SJ, Kim YS, Kwon CW, Park HK, Jeong JS, Kim JK (2009) Overexpression of the transcription factor AP37 in rice improves grain yield under drought conditions. Plant Physiology 150: 1368-1379. doi: 10.1104/pp.109.137554.

Pei LM, Wang JM, Li KP, Li YJ, Li B, Gao F, Yang AF (2012) Overexpression of thellungiella halophila H+-pyrophosphatase gene improves low phosphate tolerance in maize. Plos One 7. doi: ARTN e4350110.1371/journal.pone.0043501.

Pena PA, Quach T, Sato S, Ge Z, Nersesian N, Changa T, Dweikat I, Soundararajan M, Clemente TE (2017a) Expression of the Maize Dof1 Transcription Factor in Wheat and Sorghum. Frontiers in Plant Science 8: 434. doi: 10.3389/fpls.2017.00434.

Pena PA, Quach T, Sato S, Ge Z, Nersesian N, Dweikat IM, Soundararajan M, Clemente T (2017b) Molecular and phenotypic characterization of transgenic wheat and sorghum events expressing the barley alanine aminotransferase. Planta 246: 1097-1107. doi: 10.1007/s00425-017-2753-1.

Peng CJ, Xu WG, Hu L, Li Y, Qi XL, Wang HW, Hua X, Zhao MZ (2018) Effects of the maize C-4 phosphoenolpyruvate carboxylase (ZmPEPC) gene on nitrogen assimilation in transgenic wheat. Plant Growth Regulation 84: 191-205. doi: 10.1007/s10725-017-0332-x.

Petzold-Maxwell JL, Meinke LJ, Gray ME, Estes RE, Gassmann AJ (2013) Effect of Bt maize and soil insecticides on yield, injury, and rootworm survival: implications for resistance management.

Journal of Economic Entomology 106: 1941-1951. doi: 10.1603/ec13216.

Qin KZ, Qiu P, Wen JY, Zhu YG, Li NW, Li SQ (2016a) High throughput transformation of a Sorghum cDNA library for rice improvement. Plant Cell Tissue and Organ Culture 125: 471-478. doi: 10.1007/s11240-016-0962-0.

Qin N, Xu WG, Hu L, Li Y, Wang HW, Qi XL, Fang YH, Hua X (2016b) Drought tolerance and proteomics studies of transgenic wheat containing the maize C-4 phosphoenolpyruvate carboxylase (PEPC) gene. Protoplasma 253: 1503-1512. doi: 10.1007/s00709-015-0906-2.

Qu B, He X, Wang J, Zhao Y, Teng W, Shao A, Zhao X, Ma W, Wang J, Li B, Li Z, Tong Y (2015) A wheat CCAAT box-binding transcription factor increases the grain yield of wheat with less fertilizer input. Plant Physiology 167: 411-423. doi: 10.1104/pp.114.246959.

Rahman H, Ramanathan V, Nallathambi J, Duraialagaraja S, Muthurajan R (2016) Over-expression of a NAC 67 transcription factor from finger millet (Eleusine coracana L.) confers tolerance against salinity and drought stress in rice. BMC Biotechnology 16. doi: 10.1186/s12896-016-0261-1.

Redillas MCFR, Jeong JS, Kim YS, Jung H, Bang SW, Choi YD, Ha SH, Reuzeau C, Kim JK (2012) The overexpression of OsNAC9 alters the root architecture of rice plants enhancing drought resistance and grain yield under field conditions. Plant Biotechnology Journal 10: 792-805. doi: 10.1111/j.1467-7652.2012.00697.x.

Saalbach I, Mora-Ramirez I, Weichert N, Andersch F, Guild G, Wieser H, Koehler P, Stangoulis J, Kumlehn J, Weschke W, Weber H (2014) Increased grain yield and micronutrient concentration in transgenic winter wheat by ectopic expression of a barley sucrose transporter. Journal of Cereal Science 60: 75-81. doi: 10.1016/j.jcs.2014.01.017.

Sadumpati V, Kalambur M, Vudem DR, Kirti PB, Khareedu VR (2013) Transgenic indica rice lines, expressing Brassica juncea Nonexpressor of pathogenesis-related genes 1 (BjNPR1), exhibit enhanced resistance to major pathogens. Journal of Biotechnology 166: 114-121. doi: 10.1016/j.jbiotec.2013.04.016.

Saeng-ngam S, Takpirom W, Buaboocha T, Chadchawan S (2012) The role of the OsCam1-1 salt stress sensor in ABA accumulation and salt tolerance in rice. Journal of Plant Biology 55: 198-208. doi: 10.1007/s12374-011-0154-8.

Selvaraj MG, Valencia MO, Ogawa S, Lu YZ, Wu LY, Downs C, Skinner W, Lu ZJ, Kridl JC, Ishitani M, van Boxtel J (2017) Development and field performance of nitrogen use efficient rice lines for Africa. Plant Biotechnology Journal 15: 775-787. doi: 10.1111/pbi.12675.

Sen P, Ghosh S, Sarkar SN, Chanda P, Mukherjee A, Datta SK, Datta K (2017) Pyramiding of three C4 speciic genes towards yield enhancement in rice. Plant Cell Tissue and Organ Culture 128: 145-160.

Shen Y, Li SJ, Jiang Z, Ma LL, Lin HJ, Peng HW, Liu L, Yuan GS, Zhao MJ, Gao SB, Zhang ZM, Pan GT (2012) Overexpression of an Incw2 gene in endosperm improved yield-related traits in maize. Maydica 57: 147-153.

Shrawat AK, Carroll RT, DePauw M, Taylor GJ, Good AG (2008) Genetic engineering of improved nitrogen use efficiency in rice by the tissue-specific expression of alanine aminotransferase. Plant Biotechnology Journal 6: 722-732. doi: 10.1111/j.1467-7652.2008.00351.x.

Shu QY, Cui HR, Ye GY, Wu DX, Xia YW, Gao MW, Altosaar I (2002) Agronomic and morphological characterization of Agrobacterium-transformed Bt rice plants. Euphytica 127: 345-352. doi: 10.1023/a:1020358617257.

Singer JW, Heckman JR, Ingerson-Mahar J, Westendorf ML (2000) Hybrid and nitrogen source affect yield and European corn borer damage. Journal of Sustainable Agriculture 16: 5-15. doi: 10.1300/J064v16n01_03.

Song ZZ, Yang SY, Zuo J, Su YH (2014) Over-expression of ApKUP3 enhances potassium nutrition and drought tolerance in transgenic rice. Biologia Plantarum 58: 649-658. doi: 10.1007/s10535-014-0454-1.

Tefera T, Mugo S, Mwimali M, Anani B, Tende R, Beyene Y, Gichuki S, Oikeh SO, Nang'ayo F, Okeno J, Njeru E, Pillay K, Meisel B, Prasanna BM (2016) Resistance of Bt-maize (MON810) against the stem borers Busseola fusca (Fuller) and Chilo partellus (Swinhoe) and its yield performance in Kenya. Crop Protection 89: 202-208. doi: 10.1016/j.cropro.2016.07.023.

Traore SB, Carlson RE, Pilcher CD, Rice ME (2000) Bt and non-Bt maize growth and development as affected by temperature and drought stress. Agronomy Journal 92: 1027-1035. doi: 10.2134/agronj2000.9251027x.

Tripathy MK, Tiwari BS, Reddy MK, Deswal R, Sopory SK (2017) Ectopic expression of PgRab7 in rice plants (Oryza sativa L.) results in differential tolerance at the vegetative and seed setting stage during salinity and drought stress. Protoplasma 254: 109-124. doi: 10.1007/s00709-015-0914-2.

Voorend W, Nelissen H, Vanholme R, De Vliegher A, Van Breusegem F, Boerjan W, Roldan-Ruiz I, Muylle H, Inze D (2016) Overexpression of GA20-OXIDASE1 impacts plant height, biomass allocation and saccharification efficiency in maize. Plant Biotechnology Journal 14: 997-1007. doi: 10.1111/pbi.12458.

Wan BL, Zha ZP, Li JB, Xia MY, Du XS, Lin YJ, Yin DS (2014) Development of elite rice restorer lines in the genetic background of R022 possessing tolerance to brown planthopper, stem borer, leaf folder and herbicide through marker-assisted breeding. Euphytica 195: 129-142. doi: 10.1007/s10681-013-0988-x.

Wang F, Jian ZP, Nie LX, Cui KH, Peng SB, Lin YJ, Huang JL (2012a) Effects of N treatments on the yield advantage of Bt-SY63 over SY63 (Oryza sativa) and the concentration of Bt protein. Field Crops Research 129: 39-45. doi: 10.1016/j.fcr.2012.01.011.

Wang F, Ye C, Zhu LY, Nie LX, Cui KH, Peng SB, Lin YJ, Huang JL (2012b) Yield differences between Bt transgenic rice lines and their non-Bt counterparts, and its possible mechanism. Field Crops Research 126: 8-15. doi: 10.1016/j.fcr.2011.09.017.

Wang J, Sun JH, Miao J, Guo JK, Shi ZL, He MQ, Chen Y, Zhao XQ, Li B, Han FP, Tong YP, Li ZS (2013) A phosphate starvation response regulator Ta-PHR1 is involved in phosphate signalling and increases grain yield in wheat. Annals of Botany 111: 1139-1153. doi: 10.1093/aob/mct080.

Wang W, Hu B, Yuan D, Liu Y, Che R, Hu Y, Ou S, Liu Y, Zhang Z, Wang H, Li H, Jiang Z, Zhang Z, Gao X, Qiu Y, Meng X, Liu Y, Bai Y, Liang Y, Wang Y, Zhang L, Li L, Sodmergen, Jing H, Li J, Chu C (2018) Expression of the Nitrate Transporter Gene OsNRT1.1A/OsNPF6.3 Confers High Yield and Early Maturation in Rice. Plant Cell 30: 638-651. doi: 10.1105/tpc.17.00809.

Wang YM, Zhang GA, Du JP, Wang MC, Liu BA (2010) Influence of transgenic hybrid rice expressing a fused gene derived from cry1Ab and cry1Ac on primary insect pests and rice yield. Crop Protection 29: 128-133. doi: 10.1016/j.cropro.2009.10.004.

Wei AY, He CM, Li B, Li N, Zhang JR (2011) The pyramid of transgenes TsVP and BetA effectively enhances the drought tolerance of maize plants. Plant Biotechnology Journal 9: 216-229. doi:

10.1111/j.1467-7652.2010.00548.x.
Wiatrak PJ, Wright DL, Marois JJ, Sprenkel R (2004) Corn - Corn hybrids for late planting in the Southeast. Agronomy Journal 96: 1118-1124. doi: 10.2134/agronj2004.1118.
Wu DM, Cao HP, Yu X, L., Shen H (2015) Aluminium tolerance of OsPIN2 overexpressed rice seedlings under pot culture. Chinese Journal of Rice Science 29: 250-258.
Wu LQ, Fan ZM, Guo L, Li YQ, Chen ZL, Qu LJ (2005) Over-expression of the bacterial nhaA gene in rice enhances salt and drought tolerance. Plant Science 168: 297-302. doi: 10.1016/j.plantsci.2004.05.033.
Xiang DJ, Man LL (2018) EhEm1, a novel Em-like protein from Eutrema halophilum, confers tolerance to salt and drought stresses in rice. Molecular Breeding 38. doi: ARTN 1710.1007/s11032-017-0750-5.
Xiao BZ, Huang YM, Tang N, Xiong LZ (2007) Over-expression of a LEA gene in rice improves drought resistance under the field conditions. Theoretical and Applied Genetics 115: 35-46. doi: 10.1007/s00122-007-0538-9.
Xie WZ, Zhao YY, Xu HM, Guo JL, Song DH, Gao Z, Xiao K (2016) Function analysis of TaMIR1129, a small molecule RNA member in wheat, in mediating plant tolerance to nitrogen deprivation. Journal of Agricultural University of Hebei 39: 12-17.
Xu MY, Li LH, Fan YL, Wan JM, Wang L (2011) ZmCBF3 overexpression improves tolerance to abiotic stress in transgenic rice (Oryza sativa) without yield penalty. Plant Cell Reports 30: 1949-1957. doi: 10.1007/s00299-011-1103-1.
Xue ZY, Zhi DY, Xue GP, Zhang H, Zhao YX, Xia GM (2004) Enhanced salt tolerance of transgenic wheat (Tritivum aestivum L.) expressing a vacuolar Na+/H+ antiporter gene with improved grain yields in saline soils in the field and a reduced level of leaf Na+. Plant Science 167: 849-859. doi: 10.1016/j.plantsci.2004.05.034.
Yang DH, Kwak KJ, Kim MK, Park SJ, Yang KY, Kang H (2014) Expression of Arabidopsis glycine-rich RNA-binding protein AtGRP2 or AtGRP7 improves grain yield of rice (Oryza sativa) under drought stress conditions. Plant Science 214: 106-112. doi: 10.1016/j.plantsci.2013.10.006.
Yang Z, Chen H, Tang W, Hua HX, Lin YJ (2011) Development and characterisation of transgenic rice expressing two Bacillus thuringiensis genes. Pest Management Science 67: 414-422. doi: 10.1002/ps.2079.
Yanni SF, Whalen JK, Ma BL (2011) Field-Grown Bt and non-Bt Corn: Yield, Chemical Composition, and Decomposability. Agronomy Journal 103: 486-493. doi: 10.2134/agronj2010.0367.
Yi KK, Wu ZC, Zhou J, Du LM, Guo LB, Wu YR, Wu P (2005) OsPTF1, a novel transcription factor involved in tolerance to phosphate starvation in rice. Plant Physiology 138: 2087-2096. doi: 10.1104/pp.105.063115.
Yu TF, Xu ZS, Guo JK, Wang YX, Abernathy B, Fu JD, Chen X, Zhou YB, Chen M, Ye XG, Ma YZ (2017) Improved drought tolerance in wheat plants overexpressing a synthetic bacterial cold shock protein gene SeCspA. Scientific Reports 7. doi: 10.1038/srep44050.
Zha XJ, Luo XJ, Qian XY, He GM, Yang MF, Li Y, Yang JS (2009) Over-expression of the rice LRK1 gene improves quantitative yield components. Plant Biotechnology Journal 7: 611-620. doi: 10.1111/j.1467-7652.2009.00428.x.
Zhang C, Li X, He YF, Zhang JF, Yan T, Liu XL (2017) Physiological investigation of C-4-phosphoenolpyruvate-carboxylase-introduced rice line shows that sucrose metabolism is

involved in the improved drought tolerance. Plant Physiology and Biochemistry 115: 328-342. doi: 10.1016/j.plaphy.2017.03.019.

Zhang F, Sun YF, Pei WX, Jain A, Sun R, Cao Y, Wu XN, Jiang TT, Zhang L, Fan XR, Chen AQ, Shen QR, Xu GH, Sun SB (2015a) Involvement of OsPht1;4 in phosphate acquisition and mobilization facilitates embryo development in rice. The Plant Journal 82: 556-569. doi: 10.1111/tpj.12804.

Zhang H, Xu W, Wang H, Hu L, Li Y, Qi X, Zhang L, Li C, Hua X (2014) Pyramiding expression of maize genes encoding phosphoenolpyruvate carboxylase (PEPC) and pyruvate orthophosphate dikinase (PPDK) synergistically improve the photosynthetic characteristics of transgenic wheat. Protoplasma 251: 1163-1173. doi: 10.1007/s00709-014-0624-1.

Zhang SJ, Li N, Gao F, Yang AF, Zhang JR (2010) Over-expression of TsCBF1 gene confers improved drought tolerance in transgenic maize. Molecular Breeding 26: 455-465. doi: 10.1007/s11032-009-9385-5.

Zhang YJ, Tan LB, Zhu ZF, Yuan LX, Xie DX, Sun CQ (2015b) TOND1 confers tolerance to nitrogen deficiency in rice. The Plant Journal 81: 367-376. doi: 10.1111/tpj.12736.

Zhao D, Derkx AP, Liu DC, Buchner P, Hawkesford MJ (2015) Overexpression of a NAC transcription factor delays leaf senescence and increases grain nitrogen concentration in wheat. Plant Biology 17: 904-913. doi: 10.1111/plb.12296.

Zhou B, Lin JZ, Peng D, Yang YZ, Guo M, Tang DY, Tan XF, Liu XM (2017) Plant architecture and grain yield are regulated by the novel DHHC-type zinc finger protein genes in rice (Oryza sativa L.). Plant Science 254: 12-21. doi: 10.1016/j.plantsci.2016.08.015.

Zhou YB, Liu H, Zhou XC, Yan YZ, Du CQ, Li YX, Liu DR, Zhang CS, Deng XL, Tang DY, Zhao XY, Zhu YH, Lin JZ, Liu XM (2014) Over-expression of a fungal NADP(H)-dependent glutamate dehydrogenase PcGDH improves nitrogen assimilation and growth quality in rice. Molecular Breeding 34: 335-349. doi: 10.1007/s11032-014-0037-z.
