# Supplementary material for: Genetically modified crops are superior in their nitrogen use efficiency-A meta-analysis of three major cereals
Source: Sci Rep. 2020 May 22;10:8568. doi: 10.1038/s41598-020-65684-9 (PMC7244766; doi:10.1038/s41598-020-65684-9)
Supplement: Supplementary file 3 — Supplementary Table 7. [file 41598_2020_65684_MOESM3_ESM.pdf]

Genetically modified crops are superior in their nitrogen use efficiency-A meta-analysis of three major cereals

Mengjiao Li [1], Jili Xu [1], Zhiyuan Gao [1], Hui Tian [1,*], Yajun Gao [1,*] & Khalil Kariman[2]

[1] Key Laboratory of Plant Nutrition and Agri-environment in Northwest China, Ministry of Agriculture, College of Natural Resources and Environment, Northwest A&F University, Yangling, Shaanxi, China

[2] School of Agriculture and Environment, The University of Western Australia, Crawley, WA 6009, Australia

Corresponding authors:

1. Hui Tian, tianh@nwsuaf.edu.cn
2. Yajun Gao, yajungao@nwafu.edu.cn

Supplementary Table 7. Gene categories involved in the collected studies

Gene name	Description	Category
Bt	Bt protein	**Biotic-stress related Protein (BSRP)**
Bar	Herbicide resistance gene	
BjNPR1	Pathogenesis-related genes 1	
PcGDH	Fungi NADP(H)-dependent GDH	**Enzyme**
ZmPti1-1	Serine/threonine kinase	
Incw2	The enzyme that mobilizes photoassimilated sucrose into numerous reactions of the developing plant seeds, associated with grain yield	
PDH45	DEAD-box helicase	
SAPK9	Sucrose non-fermenting 1-related kinase	
PEPC/PPDK	Phosphoenolpyruvate carboxylase	
appA	Phytase	
AVP1	Vacuolar H+-pyrophosphatase	
PLD	Phospholipase D	
VP	V-H^+Ppase	
LRK1	Leucine-rich repeat receptor-like kinase	
AtGA2ox8	Gibberellin 2-oxidase	
SRK	S-domain receptor-like kinase extracellular domain，involved in the self-incompatibility response	
AlaAt	Alanine amino transferase	
ASN	Asparagine synthetase	
GS1	Cytosolic glutamine synthetase isoenzymes	
ZmCIPK21	CBL-Interacting Kinase	
PsGPD	Glyceral-Dehyde-3-phosphate dehydrogenase	
SnRK	Sucrose on-fermenting-1-related protein kinase	
MDH	Mitochondrial malate dehydrogenase	
GA20-OXIDASE1	GA biosynthesis	
AVP1	Arabidopsis vacuolar H+ -pyrophosphatase	
TPP	Trehalose-6-phosphate phosphatase	
TsVP	Vacuolar H^+-pyrophosphatase	
BrECS	Gamma-glutamylcysteine	

	synthetase	
LOS5	Molybdenum cofactor sulfurase	
Cu/Zn-SOD+APX	Superoxide dismutase/ascorbate peroxidase	
SaVHAC1	A vacuolar H+ -ATPase subunit c1 gene from the halophyte grass *Spartina alterniflora* Lo ¨isel	
SARK9	Sucrose non-fermenting 1-related kinase 2 gene	
OsACS2	1-aminocyclopropane-1-carboxylic acid synthase	
OsCPK4	Calcium-dependent protein kinase	
OsHsFA2D	Heat Shock transcription factor	Transcription factor (TF)
TsCBF1	C-repeat-binding transcription factor	
NAC5, 6, 9, 67, 69	NAC transcription factor	
AP37, 59	Transcription factor	
ZmDOF1	DOF transcription factor	
OsMYB55	MYB transcription factor	
CCAAT Box-Binding Transcription Factor	CCAAT Box-Binding transcription Factor	
OsPTF1	Transcription factor	
DREB1A	Encodes a member of the DREB subfamily A-1 of ERF/AP2 transcription factor family (CBF3)	
DHHC-type	DHHC-type zinc finger protein	
Ossta2	Oryza sativa salt tolerance activation 2-Dominant	
W16	AP2/EREBP transcription factor	
DHHC	Zinc-finger protein	
OsHAK1	High-affinity potassium transporter	Transporter
NAT3	A nitrate transporter of cylindrotheca fusiformis	
OsPTR6	Nitrate transporter	
STP13	Hexose transporter	
ApKUP3	High-affinity potassium transporters	
PHO1	Pi transporter	
PgNHX1	Pennisetum glaucum vacuolar Na^+/H^+ antiporter gene	

nhaA	Na^+/H^+ antiporter	
HvSUT1	Sucrose transporter	
OsNRT2.3b/OsNPF7.3/OsNRT1.1A.OsNPF6.3/OsNRT2.1/OsNPF7.9/OsNRT1.2	Nitrate transporter	
OsAMT1-1	Ammonium transporter	
HVA1	Late embryogenesis abundant protein	
AISAP	Stress associated protein	
OsEOD93-1	Nitrogen-responsive early nodulin gene	
Tin	Tiller inhibition gene	
OCP11	Stress responsive proteinase inhibitor gene	
Bn-csRRM2	A conserved RNA-binding motif (RRM) domain，flowering control locus A (FCA) protein	
TaPHR1	A phosphate starvation response regulator	
LEA	Late embryogenesis abundant	
SeCspA	Synthetic bacterial cold shock protein gene	
TOND1	Tolerance of nitrogen deficiency	
OsPIN2	Efflux-facilitating PIN-FORMED	
TaMIR1129	miRNA	Other genes
OsDhn1	Dehydrin gene	
OsCam1-1	Salt-inducible calmodulin gene	
RAA1	Root architecture associated 1	
MoSM1	Immunity-inducing protein	
SaARF1	ADP ribosylation factor	
RPL23A	Ribosomal protein large	
PgRab7	Vesicle trafficking protein	
AtGRP2	Glycine-rich RNA-binding protein	
SeCspA	Synthetic bacterial cold shock protein	
U	Unknown genes	
EhEM1	Em-like protein from Eutrema halophilum	
OsENOD93-1	A nitrogen-responsive early nodulin	

	gene
OsPGIP1	Polygalacturonase-inhibiting proteins
