# Supplementary material for: Genetically modified crops are superior in their nitrogen use efficiency-A meta-analysis of three major cereals
Source: Sci Rep. 2020 May 22;10:8568. doi: 10.1038/s41598-020-65684-9 (PMC7244766; doi:10.1038/s41598-020-65684-9)
Supplement: Supplementary file 4 — Supplementary Table 8. [file 41598_2020_65684_MOESM4_ESM.pdf]

Genetically modified crops are superior in their nitrogen use efficiency-A meta-analysis of three major cereals

Mengjiao Li [1], Jili Xu [1], Zhiyuan Gao [1], Hui Tian [1,*], Yajun Gao [1,*] & Khalil Kariman[2]

[1] Key Laboratory of Plant Nutrition and Agri-environment in Northwest China, Ministry of Agriculture, College of Natural Resources and Environment, Northwest A&F University, Yangling, Shaanxi, China

[2] School of Agriculture and Environment, The University of Western Australia, Crawley, WA 6009, Australia

Corresponding authors:

1. Hui Tian, tianh@nwsuaf.edu.cn
2. Yajun Gao, yajungao@nwafu.edu.cn

Supplementary Table 8. Summary of results from the meta-analyses on yield, shoot biomass, shoot N utilization efficiency (SNUE), N uptake efficiency (NUpE), grain N utilization efficiency (GNUE), and partial factor productivity of N (PFPN) by using the complete dataset and by using one random observation per study.

**Parameters**	**Dataset**	***n***	**Log$_e$*R* (%)**	**95% CI**		***P***
				**Min.**	**Max.**	
Yield	Complete dataset	870	0.1546	0.1289	0.1804	< 0.001
	One random observation per study	109	0.1663	0.0832	0.2493	< 0.001
Shoot biomass	Complete dataset	533	0.0960	0.0583	0.1336	< 0.001
	One random observation per study	52	0.1582	0.0206	0.2957	< 0.05
SNUE	Complete dataset	107	-0.0682	-0.0906	-0.0459	< 0.001
	One random observation per study	17	-0.0925	-0.1630	-0.0220	< 0.05
NUpE	Complete dataset	193	0.1510	0.1139	0.1882	< 0.001
	One random observation per study	23	0.2430	0.1133	0.3727	< 0.001
YNUE	Complete dataset	53	-0.0517	-0.0890	-0.0143	< 0.01
	One random observation per study	11	-0.0878	-0.1655	-0.01	< 0.05
PFPN	Complete dataset	268	0.0905	0.0632	0.1179	< 0.001
	One random observation per study	29	0.1035	0.0293	0.1776	< 0.01
