# Supplementary material for: Genetically modified crops are superior in their nitrogen use efficiency-A meta-analysis of three major cereals
Source: Sci Rep. 2020 May 22;10:8568. doi: 10.1038/s41598-020-65684-9 (PMC7244766; doi:10.1038/s41598-020-65684-9)
Supplement: Supplementary file 6 — Supplementary Fig. 1. [file 41598_2020_65684_MOESM6_ESM.pdf]

Genetically modified crops are superior in their nitrogen use efficiency-A meta-analysis of three major cereals

Mengjiao Li [1], Jili Xu [1], Zhiyuan Gao [1], Hui Tian [1,*], Yajun Gao [1,*]& Khalil Kariman[2]

[1] Key Laboratory of Plant Nutrition and Agri-environment in Northwest China, Ministry of Agriculture, College of Natural Resources and Environment, Northwest A&F University, Yangling, Shaanxi, China

[2] School of Agriculture and Environment, The University of Western Australia, Crawley, WA 6009, Australia

Corresponding authors:

1. Hui Tian, tianh@nwsuaf.edu.cn
2. Yajun Gao, yajungao@nwafu.edu.cn

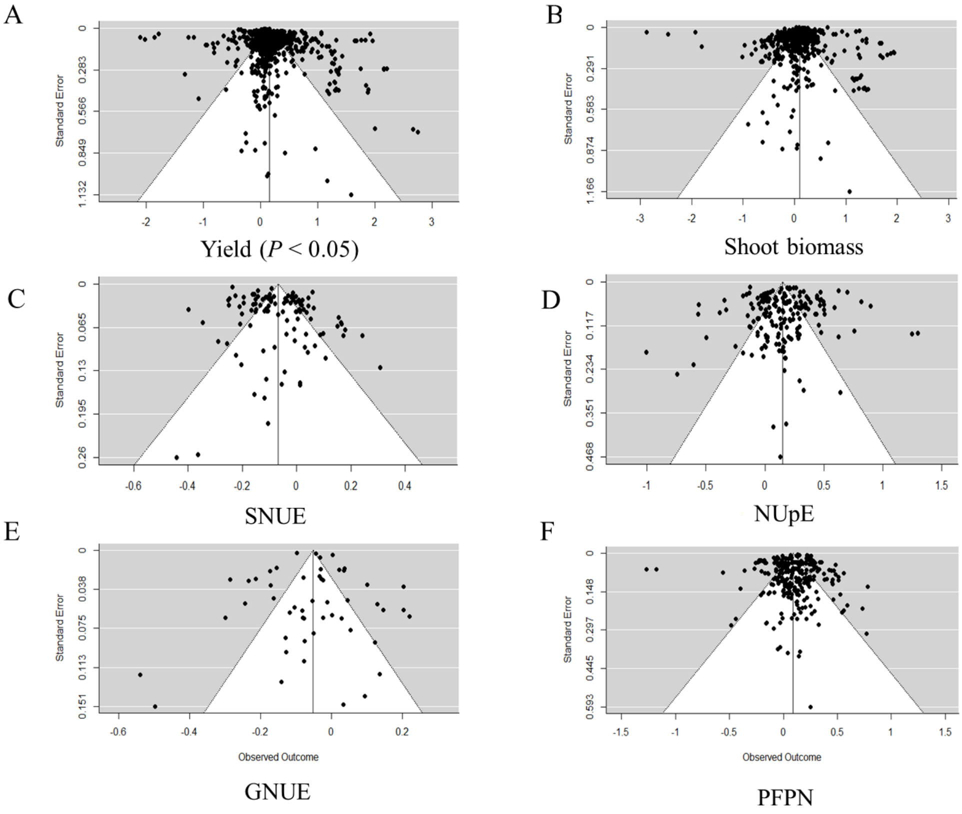
A
B
C
D
E
F
Standard Error
Yield ($P < 0.05$)
Shoot biomass
SNUE
NUpE
Observed Outcome
GNUE
PFPN
